# The role of upper and lower genital tract microbiota alterations in term chorionamnionitis: A prospective study

**DOI:** 10.3389/fmicb.2022.1069254

**Published:** 2022-12-20

**Authors:** Meng Li, Zhenyu Huang, Zhi Tao, Yiting Meng, Jia Wen, Qiongqiong Zhang, Ying Liu, Mengyuan Shang, Ying Wang, Yufeng Wang, Rui Chen, Xiaoqian Wang, Yang Cao, Lei Zhang, Qinping Liao

**Affiliations:** ^1^School of Clinical Medicine, Tsinghua University, Beijing, China; ^2^Department of Obstetrics and Gynecology, Beijing Tsinghua Changgung Hospital, School of Clinical Medicine, Tsinghua University, Beijing, China

**Keywords:** pregnancy, chorioamnionitis, microbiome, infection, inflammation

## Abstract

**Objective:**

This study aimed to compare the dynamics of lower and upper genital tract microbiota in normal term pregnancy, histological chorioamnionitis (HCA), and clinical chorioamnionitis (CCA) patients to provide a reference for the diagnosis and treatment of chorioamnionitis (CAM) patients.

**Methods:**

We prospectively collected vaginal and cervical secretions, as well as placenta tissues, fetal membranes, and amniotic fluid from normal-term pregnant women, HCA and CCA patients. Then, we performed genomic DNA extraction and PCR amplification for all samples. The eligible samples were analyzed by 16S ribosomal RNA (16S rRNA) sequencing. Additionally, all placenta tissues were histopathologically examined, and neonatal pharyngeal swabs and placenta tissues from the HCA and CCA groups were subjected to microbial culture.

**Results:**

A total of 85 term pregnant women were enrolled in this study, including 34 in the normal group (N), 37 in the HCA group, and 14 in the CCA group. A total of 171 qualified samples were analyzed by 16S rRNA sequencing. The results suggested that the cervical microbiota was highly similar to the vaginal microbiota in normal term parturients, with *Lactobacillus* as the dominant bacterium. Moreover, there was no difference in the alpha and beta diversity of vaginal microbiota between the N, HCA, and CCA groups at the genus level. Besides, no significant differences were detected in cervical microbiome among the three groups. Regarding intrauterine microorganisms, the N and HCA groups had similar microbial composition but were different from the CCA group. No microbe was detected in the placental tissue of normal term parturients, while some microorganisms were found in the intrauterine amniotic fluid and fetal membrane samples. Regardless of cultivation or 16S rRNA sequencing, an extremely low microbial positive rate was detected in HCA and CCA intrauterine samples. Compared to the normal group, *Lactobacillus* was significantly reduced in the CCA group intrauterine, and *Ureaplasma* and *Enterococcus* increased with no statistically significant.

**Conclusion:**

The N, HCA and CCA groups had similar composition of vaginal and cervical microflora. Some normal-term pregnant women can harbor non-pathogenic microbiota in the uterine cavity. Sterile inflammation is more frequent than microbial-associated inflammation in term HCA and CCA parturients.

## Introduction

Chorioamnionitis (CAM) or intraamniotic infection is a common pregnancy complication. It comprehends an inflammation or infection of the fetal membrane, amniotic fluid, placenta, umbilical cord, or any part of the fetus (Committee on Obstetric Practice, 2017). According to the existence of clinical manifestations, CAM is divided into clinical chorioamnionitis (CCA) and histological chorioamnionitis (HCA; Beck et al., 2021). Additionally, CCA is characterized by the following clinical symptoms: maternal fever, uterine tenderness, maternal and/or fetal tachycardia, maternal leukocytosis, and foul-smelling or purulent amniotic fluid (Tita and Andrews, 2010). Moreover, HCA (also known as subclinical chorioamnionitis) is more common than CCA and is defined by diffuse infiltration of neutrophils into the chorioamniotic membranes (Levy et al., 2021).

Traditionally, it was assumed that the healthy uterus and intrauterine environment (placenta, chorion/amnion, amniotic fluid, fetus, and meconium) were sterile during pregnancy, and CAM was often associated with bacterial invasion. Currently, the concept of the “sterile womb” means that microbes can be acquired both vertically (from the mother) and horizontally (from other humans or the environment) during and after birth (Funkhouser and Bordenstein, 2013). However, due to the development of high-throughput sequencing technology, this view has been unprecedentedly challenged. First, Aagaard et al. (2014) have demonstrated that the placenta of healthy pregnant women harbors a unique low-abundance placental microbiome by 16S ribosomal DNA and whole-genome shotgun (WGS) metagenomic sequencing. Further, many studies have challenged the sterility of the intrauterine environment during pregnancy (Seferovic et al., 2019; Stinson et al., 2019; Ennamorati et al., 2020; Hockney et al., 2020; Rackaityte et al., 2020; Mishra et al., 2021). For example, Tuominen et al. (2019) have shown that the neonatal oral cavity microbiota seems to share common characteristics with the microbes in the placenta but not the birth canal, indicating that the neonatal oral microbiota might have a prenatal origin. Moreover, Rackaityte et al. (2020) studied meconium samples in the mid-gestation and found that 18 taxa were enriched in the fetal meconium, especially *Micrococcaceae* and *Lactobacillus*. This view is supported by Mishra et al. (2021) who identified several live bacterial strains including *Staphylococcus* and *Lactobacillus* in fetal tissues, which induced *in vitro* activation of memory T cells in fetal mesenteric lymph node, supporting the role of microbial exposure in fetal immune initiation. Whereas, it is debated whether the microbiome detected during reproduction, especially low-biomass microbial communities, are in fact merely a result of contamination and an artifact of the study design (Lauder et al., 2016; Kim et al., 2017). Besides, increasing studies have shown a high incidence of sterile inflammation, that is, when microorganisms cannot be detected, in preterm or term CAM patients. Thus, the expert panel of the National Institute of Child Health and Human Development (NICHD) has proposed a descriptive term: “intrauterine inflammation or infection or both,” abbreviated as “Triple I,” to replace chorioamnionitis (Higgins et al., 2016). Romero et al., (2014b) analyzed amniotic fluid samples from 135 premature women with intact membranes and demonstrated that sterile intra-amniotic inflammation is more frequent than microbial-associated inflammation. Additionally, a retrospective cohort study has suggested that 24% of preterm CCA patients had no evidence of intraamniotic infection or inflammation, and 66% had negative amniotic fluid cultures (Oh et al., 2017).

A better understanding of the composition and alterations of the upper and lower genital tract microbiome might help develop better treatment strategies for CAM either in maternal or infants. Therefore, this study aimed to: (a) Quantify the bacterial load and diversity in the vagina, cervix, and uterine cavity of normal term parturients and (b) compare the microbiota composition of the upper and lower genital tracts of normal, HCA, and CCA in term pregnancy.

## Materials and methods

### Study design and enrolment

We prospectively collected vaginal and cervical secretions, as well as placenta tissue, fetal membranes, and amniotic fluid from term pregnant women who delivered at Beijing Tsinghua Changgung Hospital between March 1 and September 30, 2019. Before beginning the study, ethical clearance was granted by the Ethics Committee of Beijing Tsinghua Changgung Hospital (16121-0110). The inclusion and exclusion criteria are presented in Table 1. According to the clinical manifestations and histopathological examination results, patients were divided into the normal pregnancy (N), CCA, and HCA groups. Simultaneously, we collected clinical information from electronic medical records, mainly including maternal age, body mass index (BMI), weight gain during labor, fetal heart rate (FHR), maternal peripheral blood white blood cells (WBC), and C-reactive protein (CRP), birth weight, and neonatal infection.

**Table 1:** Inclusion and exclusion criteria.

Inclusion	Exclusion
Singleton pregnant women delivered at term in our hospital	Pregnant women who do not receive systematic health care and delivery
Intact membranes before delivery	Premature delivery
No sexual life history within 3 d before delivery	Multiple pregnancies
No history of external vaginal drugs and systemic antibiotics within 2 weeks	Premature rupture of membranes
No infection in other parts of the body	Refuse histopathological examination of the placenta
Agree to participate in the study and sign the informed consent	Infection of other parts of the body

### Sample collection

Before planning induced labor or cesarean sections, samples were taken with the patient’s consent. Vaginal secretions were obtained from the lateral wall of the upper 1/3 of the vagina, and a cervical swab was taken from 2 to 3 cm inside the cervical canal. All intrauterine tissue samples were collected following strict and uniform protocols established before the study begins using standard operating procedures by a specially trained physician. During the operation, samples were taken immediately after delivery from participants, and strict aseptic operation was performed. Then, samples were placed in sterile ice-cold phosphate-buffered saline solution (1 × PBS) and stored at −80°C within 1 h. About 2–5 ml of amniotic fluid was absorbed from the neonatal oropharynx in a sterile tube for subsequent experiments.

### Placental histopathologic examination

At the same time, another placental tissue was collected, fixed with 10% neutral buffered formalin, embedded in paraffin wax, and stained with hematoxylin and eosin (H&E) for histopathological examination. The diagnosis of pathological sections was assessed by two gynecological/placental histopathologists without knowing the clinical data. As previously mentioned, HCA staging was based on the area of neutrophil infiltration in the tissue anatomy, and the grading was based on the degree of neutrophil infiltration in a particular area (Zheng et al., 2013). Simply speaking, staging is based on the area of neutrophil infiltration in tissue anatomy, and grading is based on the degree of neutrophil infiltration in a particular area.

### Bacterial cultures

Neonatal oropharyngeal swabs and placental grind fluid were inoculated on Columbia blood agar plates using the four-zone method. Then, blood plates were cultured in aerobic and anaerobic environments for 18–24 h, respectively. Colonies were identified by matrix-assisted laser desorption ionization time-of-flight mass spectrometry (MALDI-TOF MS).

### Genomic DNA extraction and PCR amplification

Microbial community genomic DNA was extracted from above mentioned samples (vaginal and cervical secretions, placenta, fetal membrane and amniotic fluid) using the TIANamp Bacteria DNA Kit (TIANGEN BIOTECH, Beijing, China) according to manufacturer’s instructions. The DNA extract was checked on 1% agarose gel, and DNA concentration and purity were determined with NanoDrop 2000 UV–vis spectrophotometer (Thermo Scientific, Wilmington, USA). Then, the hypervariable region V3-V4 of the bacterial 16S rRNA gene were amplified with primer pairs 338F(5’-ACTCCTACGGGAGGCAGCAG-3′) and 806R(5’-GGACTACHVGGGTWTCTAAT-3′) by an ABI GeneAmp® 9,700 PCR thermocycler (ABI, CA, USA). The PCR product was extracted from 2% agarose gel and purified using the AxyPrep DNA Gel Extraction Kit (Axygen Biosciences, Union City, CA, USA) according to manufacturer’s instructions and quantified using Quantus™ Fluorometer (Promega, USA). All the amplification products were stored at −20°C for later sequencing.

### Illumina read data processing and analysis

Purified amplicons were pooled in equimolar and paired-end sequenced on an Illumina MiSeq PE300 platform/NovaSeq PE250 platform (Illumina, San Diego, USA) according to the standard protocols by Majorbio Bio-Pharm Technology Co. Ltd. (Shanghai, China). The raw 16S rRNA gene sequencing reads were demultiplexed, quality-filtered by fastp version 0.20.0 (Chen et al., 2018) and merged by FLASH version 1.2.7 (Magoč and Salzberg, 2011). Operational taxonomic units (OTUs) with 97% similarity cutoff (Stackebrandt and Goebel, 1994; Edgar, 2013) were clustered using UPARSE version 7.1 (Edgar, 2013), and chimeric sequences were identified and removed. The taxonomy of each OTU representative sequence was analyzed by RDP Classifier version 2.2 (Wang et al., 2007) against the 16S rRNA database (e.g. Silva v138) using confidence threshold of 0.7.

### Bioinformatics and statistical analysis

SPSS 23.0 (IBM Corporation, USA) software and R (v. 3.5.1) was used to process data, measurement data were expressed as mean ± standard deviation (*x* ± *s*), paired sample means were compared using paired *T* test, multiple sample means were compared by one-way ANOVA, and measurement data were compared using *χ*^2^ test. *p* < 0.05 was considered statistically significant. Biology statistical methods using multivariate statistical analysis, including: analysis of variance, rank and inspection and ternary phase diagram.

## Results

### Patient characteristics

A total of 85 term pregnant women were enrolled in this study, including 34 in the N group, 37 in the HCA group, and 14 in the CCA group. The average age of pregnant women was 30.08 ± 2.77 years old, and the average gestational age was 39.7 ± 1.0 weeks. At the end of the research, 85 neonates (42 males and 43 females) were delivered without neonatal asphyxia or perinatal death. Clinical characteristics are shown in Table 2. No differences were identified between groups regarding age, maternal weight gain, number of deliveries, and birth weight. It is apparent from Table 2 that the gestational age of the N group was smaller than the CCA and HCA groups, and the BMI of the CCA group was higher compared to other groups. Closer inspection of the table shows the WBC, CRP of pregnant women, fetal distress, neonatal infection, and transfer rate to the neonatal department were also higher in the CCA group compared to the N and HCA groups, and there was no significant difference between N and HCA group.

**Table 2:** Characteristics of participants.

	*N* (34)	HCA (37)	CCA (14)	*P*
Age, mean ± SD (*y*)	30.56 ± 3.11	29.81 ± 2.62	29.64 ± 2.27	NS
Body mass index, median ± SD (kg/m^2^)	21.54 ± 4.83	21.73 ± 2.92	24.69 ± 3.66	<0.05 ac
Weight gain in pregnancy (kg)	14.09 ± 5.17	13.83 ± 3.90	12.82 ± 4.87	NS
Primiparous women, *n* (%)	25 (73.53%)	32 (86.49%)	14 (100%)	NS
Gestational age (weeks) ± SD	39.4 ± 1.1	39.9 ± 0.8	40.1 ± 0.8	<0.05ab
Fetal distress *n* (%)	2 (5.9%)	2 (8.1%)	9 (64.3%)	<0.05 ac
Birth weight, *g* ± SD	3439.26 ± 457.23	3451.35 ± 353.54	3584.64 ± 326.01	NS
Transfer to neonatal department, *n* (%)	0 (0.0%)	3 (8.1%)	7 (50.0%)	<0.05 ac
Neonatal infection, *n* (%)	0 (0.0%)	1 (2.7%)	7 (50.0%)	<0.05 ac
Maternal CRP (mg/L)	8.51 ± 12.89	12.93 ± 13.94	20.26 ± 11.41	<0.05a
Maternal WBC (*109/L)	9.43 ± 3.47	10.92 ± 4.27	13.45 ± 2.82	<0.05 ac

a, N compared to CCA. b, N compared to HCA; c, HCA compared to CCA.

### Pathological examination

**Figure 1:**
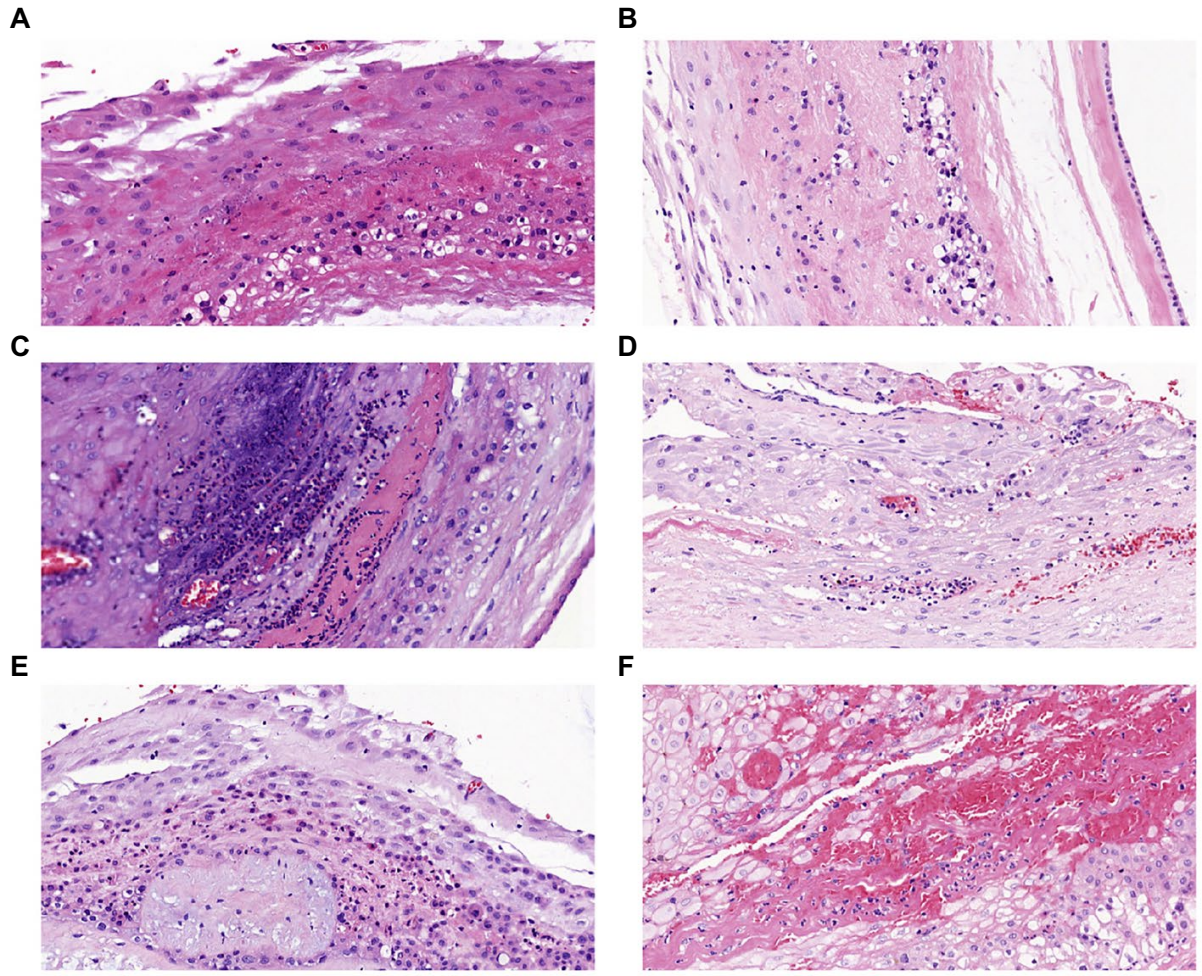
Placental histopathology chorioamnionitis (H&E × 200): **(A)** Stage I grade I, **(B)** Stage I grade II, **(C)** Stage I grade III, **(D)** Stage II grade I, **(E)** Stage II grade II, **(F)** Stage II grade III.

All the placentas of 85 pregnant women were sent for histopathological examination. Partial pathological results were shown in Figure 1, and there were no stage III patients were detected in the HCA and CCA groups. In HCA group, 21 patients diagnosed stage I and 16 patients diagnosed stage II. Interestingly, there were 4 patients had normal placental pathology in CCA group. Of the remaining 10 patients, 9 were stage II and 1 was stage I.

### Microbiologic cultures

In the HCA group, the negative rate of neonatal pharyngeal swabs was 83.3% (5/6), and no bacteria could be cultured from placenta tissues (0/11), and *Escherichia coli* and *Enterococcus faecalis* were detected in positive pharyngeal specimens. Next, in the CCA group, the negative rate for neonatal specimens was 58.3% (7/12) and for placental tissues was 64.3% (9/14). Similar to the HCA group, except the above two bacteria, the CCA group also cultured *Streptococcus agalactiae*, *Enterococcus faecium* and *Corynebacterium*.

### Analysis of 16S rRNA sequencing

A total of 85 × 5 (425) samples were collected during the research, of which 171 were qualified (few of them detected with bacterial DNA) for 16S rRNA sequencing (Table 3). The optimized sequence number was 8,678,452, the total number of bases was 3,709,462,018, and the average length was 427 bp, which were included in subsequent analysis. Herein, we analyzed the samples of placentae, fetal membranes, and amniotic fluid together, which are collectively referred to as intrauterine samples (marked as Up). Additionally, vaginal and cervical specimens were abbreviated as ‘V’ and ‘C’, respectively.

**Table 3:** Distribution of eligible and qualified samples.

Sample	N (*n*, %)	HCA (*n*, %)	CCA (*n*, %)	Total (*n*, %)
Vaginal	34 (49.3%)	37 (48.8%)	14 (53.8%)	85 (49.7%)
Cervical	28 (40.6%)	25 (32.9%)	9 (34.6%)	62 (36.3%)
Placenta	0	5 (6.6%)	1 (3.8%)	6 (3.5%)
Fetal membrane	3 (4.3%)	0	0	3 (1.8%)
Amniotic fluid	4 (5.8)	9 (11.8%)	2 (7.7%)	15 (8.8%)
Total	69	76	26	171

**Figure 2:**
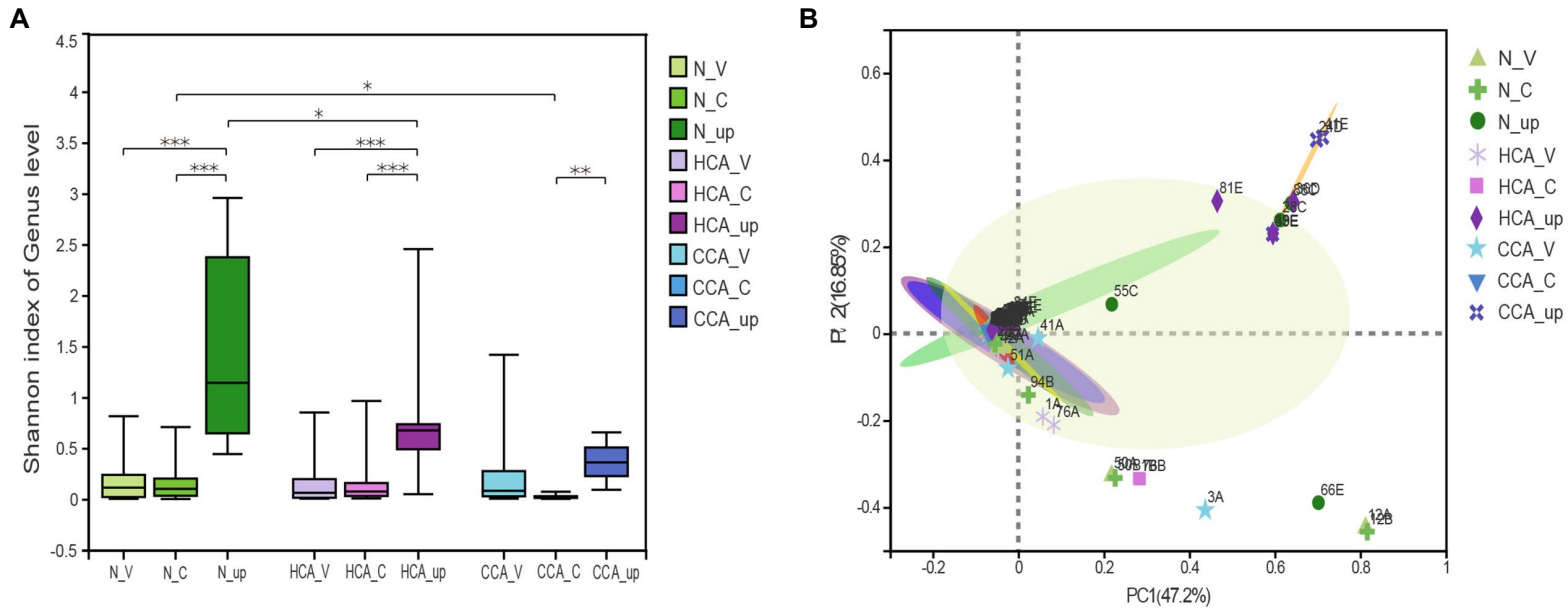
The diversity of the upper and lower genital tract microbiome were sequenced using 16S rRNA. **(A)** Alpha diversity was calculated using the Shannon diversity index in N, HCA, and CCA groups. **(B)** Beta-diversity was visualized using Principal Coordinate analysis (PCoA). The first two principal coordinate axes, which together explain 64.05% of variation, are shown. (*0.01 ≤ *p* < 0.05; **0.001 ≤ *p* < 0.01; ***0.0001 ≤ *p* < 0.001).

As can be seen from Figure 2A, the alpha diversity test (Shannon index) showed that the intrauterine bacterial diversity significantly increased compared to the vagina and cervix (*p* < 0.001). And there was no difference in the alpha diversity of vaginal microbiota between the N, HCA, and CCA groups at the genus level. Similarly, no significant differences were found between the N and HCA groups when the richness of the cervical microbiota was evaluated. The cervical microorganisms in the CCA group presented a clear downward trend compared to the N group (Figure 2A). The Principal Coordinates analysis (PCoA) showed that vaginal and cervical samples were clustered together with concentrated distribution and high similarity, while the intrauterine flora distribution was different from the other two groups with large dispersion and considerable differences, indicating that there were significant differences between intrauterine bacteria and vaginal and cervical bacteria (Figure 2B). The above groups of vaginal microbiota were clustered together and were highly similar (*p* = 0.39). Besides, no significant differences were detected in the cervical microbiome among the three groups (*p* = 0.88), indicating that there was no meaningful difference in the composition of cervical microbiome. Regarding the changes in intrauterine microorganisms, the normal and HCA groups were clustered together, while the CCA group was dispersed from the other two groups, implying that the composition of intrauterine bacteria in CCA was different from the other two groups and had its uniqueness (Figure 2B).

**Figure 3:**
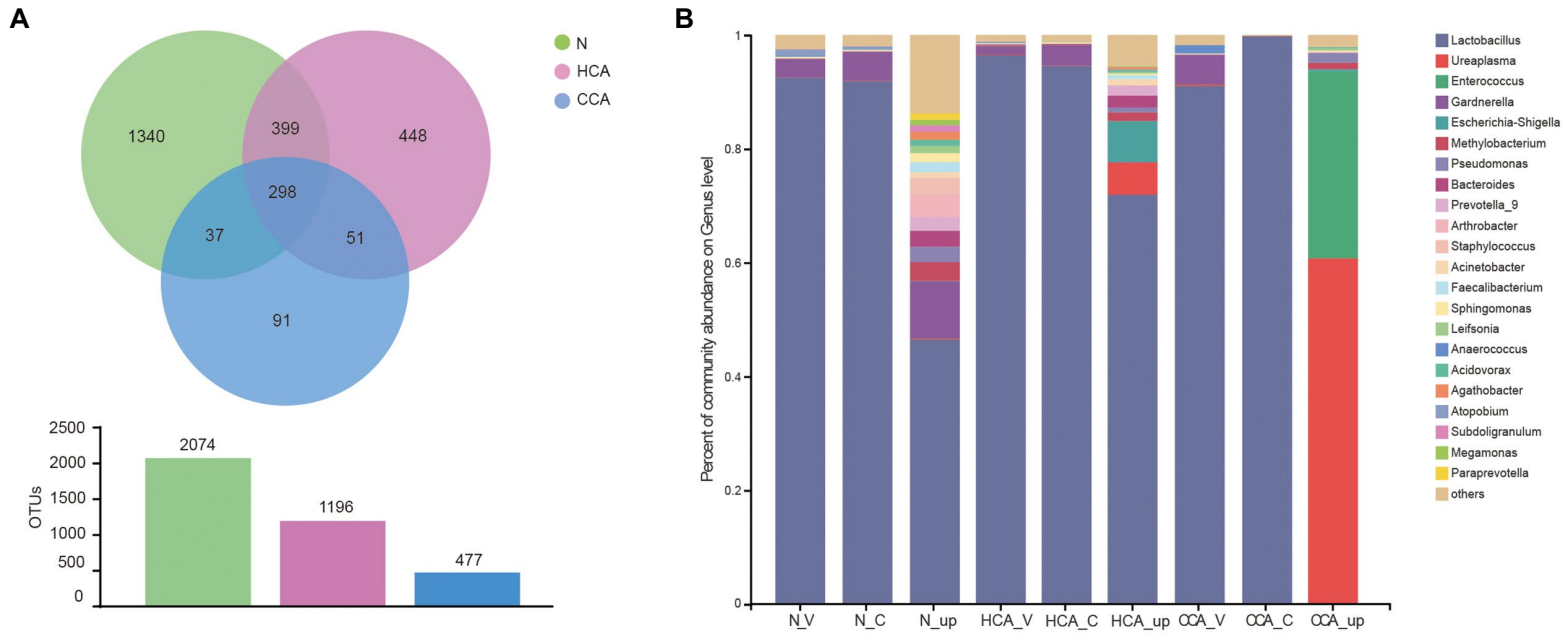
**(A)** Venn diagrams indicating the number of OTUs in the N, HCA, and CCA groups by 16S rRNA sequences; **(B)** Stacked bar graph showing the relative abundance of bacterial 16S rRNA sequences at genus level in N, HCA, and CCA groups.

Next, the number of common and unique operational taxonomic units (OTUs) among these three groups is represented by a Venn diagram (Figure 3A). The number of OTUs in the N group was the highest and decreased with the aggravation of inflammation. The vaginal and cervical microbiota in the N group were similar at the genus level and mainly composed of *Lactobacillus*, followed by *Gardnerella*, *Atopobium*, and *Streptococcus*. Additionally, a significant decrease in *Lactobacillus* was detected in the utero accompanied by an increase in *Gardnerella*, *Staphylococcus*, *Methylobacterium* and other bacteria (Figure 3B). Unlike the N and HCA groups, the intrauterine flora of the CCA group was dominated by *Ureaplasma* spp. and *Enterococcus* spp.

**Figure 4:**
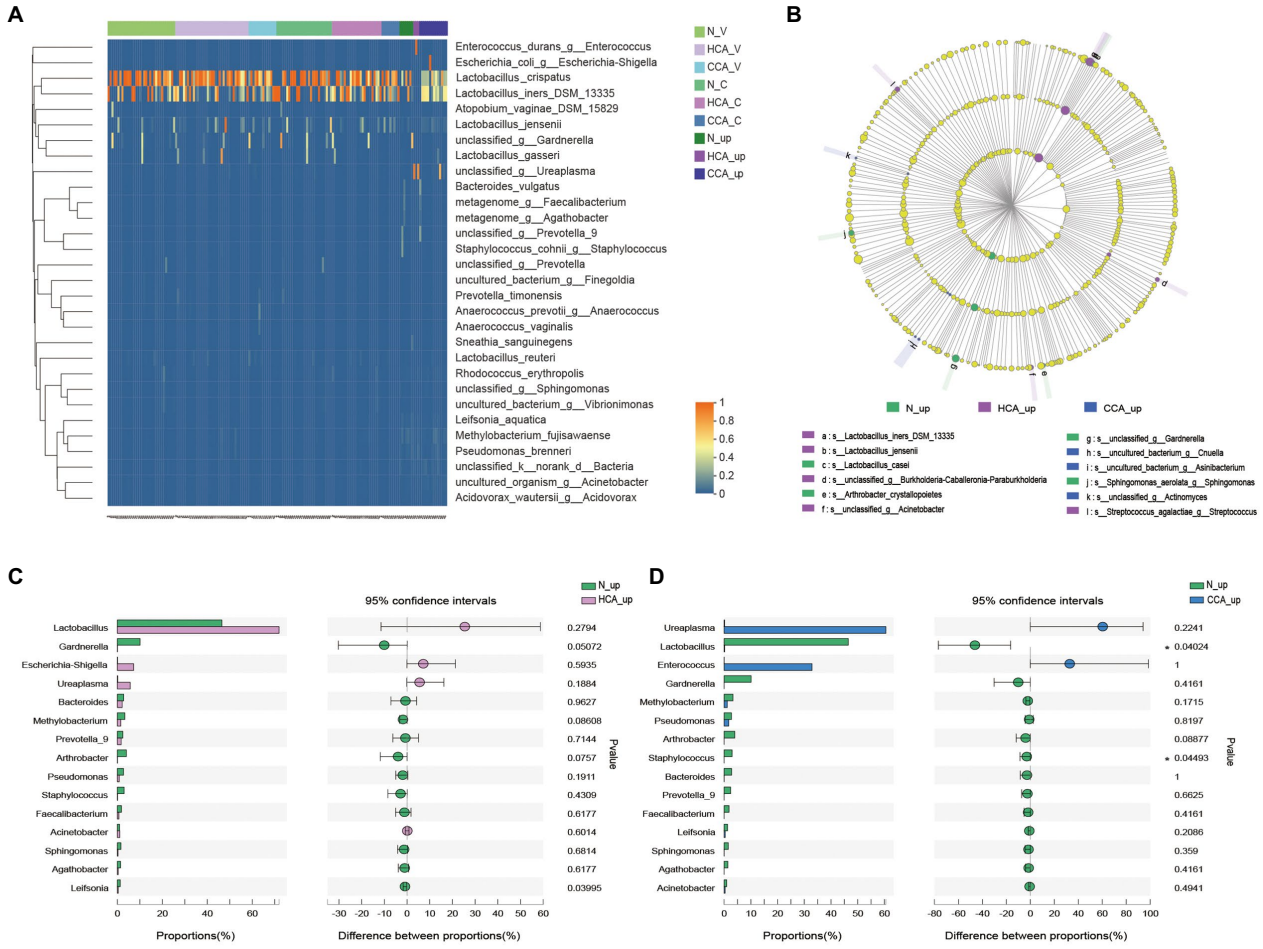
**(A)** Heatmap of upper and lower genital tract microbiota in the N, HCA, and CCA groups showing relative abundances of bacterial taxa per sample (columns). Reads were aggregated at the species level, which are shown as the different rows. **(B)** LEfSe analysis identified the microbes whose abundances significantly differed among N, HCA, and CCA groups. The findings with regards to family, genus and species are shown in the plot. Only species-level differences are shown in the legend. (Light yellow nodes indicate microbial taxa that are not significantly different in any of the different groups or have no significant effect on the differences between groups; different color nodes indicate microbial taxa that are significantly enriched in the corresponding groups and have a significant effect on the differences between groups). Analysis of intrauterine differential microbiota between N and HCA group **(C)** or between N and CCA group **(D)**. (The *X*-axis represents different subgroups, and the *Y*-axis represents the average relative abundance of a genus in different subgroups).

Further studies were conducted at the species level on lower and upper genital tract of pregnant women in these three groups by clustering heatmap analysis. The results, as shown in Figure 4A, denote that the vagina and cervix of pregnant women had the highest abundance of *L. crispatus* and *L. iners* in all groups, while *Lactobacillus* sharply decreased in the utero of HCA and CCA groups. These differences in species can also be seen in Figure 4B. In the LEfSe analysis, significant differences in intrauterine microbial abundances were observed among these three groups. *L. iners* (LDA score = 5.3, *p* = 0.027). and *L. jensenii* (LDA score = 4.2, *p* = 0.02) were over-represented in HCA group on the specises level; *Cnuella* spp. (LDA score = 4.0, *p* = 0.03) and *Asinibacterium* spp. (LDA score = 4.0, *p* = 0.03) were over-represented in CCA group on the genus level. The most interesting aspect of Figure 4B is that *Gardnerella* spp. levels were significantly higher in the uterine cavity of group N (LDA score = 4.9, *p* = 0.017). Furthermore, the Wilcoxon rank-sum test was used to analyze the intrauterine differential microbiota of each group. No significant disparity was found between the N and HCA groups (Figure 4C). Additionally, *Lactobacillus* spp. (*p* = 0.04) and *Staphylococcus* spp. (*p* = 0.04) were notably increased in the N group compared to the CCA group, while *Ureaplasma* spp. and *Enterococcus* spp. were higher in the CCA group but without no statistical significance (*p* > 0.05; Figure 4D).

## Discussion

The vaginal microbiota can be categorized into one of five major community state types (CSTs), of which four community state types are dominated by *Lactobacillus*, namely: CST-I (*L. crispatus*) CST-II (*L. gasseri*) CST-III (*L. iners*) and CST-V (*L. jensenii*; Ravel et al., 2011). Due to the substantial increasing circulating estrogen levels in pregnancy, the physicochemical environment and microecological flora of the vagina also change accordingly (Kohlhepp et al., 2018). What’s more, high estrogen status during pregnancy is associated with proliferation of vaginal epithelial cells and glycogen deposition, which is more conducive to the dominance of *Lactobacillus* (Taddei et al., 2018). Similar to non-pregnant women, the vaginal microbiota of normal pregnant women is also dominated by *Lactobacillus* spp., and this homeostasis is stable throughout whole pregnancy (Romero et al., 2014a; McMillan et al., 2015). In our research, the vaginal flora of normal term pregnant women was consistent with previous studies. This *Lactobacillus*-led physiological state can resist the invasion of pathogenic microorganisms, reduce the chance of upper genital tract infection during term delivery, and maximize protecting the health of maternal and infants (Fettweis et al., 2019; Serrano et al., 2019).

Very little was found in the literature on the research of the cervical microbiome during pregnancy. Previously published studies disclose that the cervical microbiology group is analogous to the vaginal microbiome, mainly composed of *Lactobacillus* and *Gardnerella* (Smith et al., 2012). Aagaard et al. (2012) also detected that the cervical microbiota of women in the third trimester of pregnancy may be similar to non-pregnant women. In the present study, we demonstrated that the cervical microbiota was highly similar to the vaginal microbiota in normal term parturients, with *Lactobacillus* as the dominant bacterium (Figures 3B, 4A). Meanwhile, in the alpha and beta diversity analyzes, no statistical differences were detected in the diversity and composition of vaginal and cervical microbiota. The high consistency of these two parts provided a theoretical basis for the collection of genital tract specimens of pregnant women in clinical work. That is, the relatively simple operation and low risk of upper 1/3 vaginal secretion sampling can replace cervical secretion in clinical work.

Recently, researchers have shown an increased interest in the microecology of different parts of the human body due to the development of next-generation sequencing. The two most widely used culture-independent methods are 16S rRNA amplicon and shotgun metagenomic sequencing (Malla et al., 2018). Shotgun metagenomics allows indiscriminate sequencing of all DNA material in a sample, which can provide species-level assignment, and can provide direct evidence of functional variation in genes of existing strains. In present study, 16S sequencing was selected mainly because of its cost-effective characters and higher sensitivity to samples with human cells. In addition, it is focused on the dynamics of upper and lower genital microbiota in CAM patients rather than the relationship between microbiota and function. Besides the studies on the intrauterine microbiome mentioned in the introduction, many studies have been conducted on multiple mammals and reconfirmed the existence of a microbiome in the placenta (Younge et al., 2019; Yu et al., 2019; Zakošek Pipan et al., 2020; Bi et al., 2021). The debate about whether the intrauterine environment is sterile has gained prominence with many arguing that bacterial DNA contamination from sampling sites, clinical or laboratory environments, as well as reagents and consumables, can greatly influence the results of microbiota studies, especially for low-biomass specimen types (Salter et al., 2014). Consequently, several investigators have examined the effects of strict controls for contamination on subsequent sequencing demonstrating a sterile intrauterine environment (Leiby et al., 2018; de Goffau et al., 2019; Kuperman et al., 2020; Olomu et al., 2020; Winters et al., 2022). Furthermore, de Goffau et al. (2021) re-analyzed the data from Rackaityte et al. (2020) and provided evidence that the identification of *Micrococcus* in fetal gut samples was contaminated and several findings were caused by an unrecognized batch effect. In our study, no microbe was discovered in the placental tissue of 34 term normal pregnant women by 16S rRNA sequencing, which is in line with the above perspective. However, some microorganisms were detected in the intrauterine amniotic fluid and fetal membrane samples in normal group, among which *Lactobacillus* spp. was still the dominant bacterium, accounting for 45.77% (Figure 3B). Apart from *Lactobacillus* spp., there are also *Gardnerella* spp., *Staphylococcus* spp., *Methylobacterium* spp. and other bacteria. It is worth noting that the gonadal hormones strongly influence the overall structure and function of vaginal microbiota. On the other hand, microbiota also affect the release and functions of hormones that modulate various physiological functions, such as the circadian rhythms (Kennaway, 2005; Sciarra et al., 2020). More importantly, numerous evidence on the correlation between the vagina microbiota and the development of infertility and Polycystic ovarian syndrome (PCOS), and the variation of its constitution might be useful marker of pregnancy outcomes (Heil et al., 2019; Venneri et al., 2022). Data from several studies suggest that gut dysbiosis and circadian rhythm disorder are associated with obesity, metabolic syndrome and inflammatory bowel disease. However, the formation mechanism of gut microbial rhythm, the dynamic dialog between microbes and the host remain unclear and require further investigation (Samuelson et al., 2019; Wang et al., 2022).

Herein, the inflammatory indexes CRP and WBC were significantly increased in the CCA group, as well as the incidence of abnormal FHR monitoring, while no difference was detected between the HCA and normal groups. Moreover, the proportion of neonatal transfer rate and incidence of neonatal infection was notably higher in the CCA group than in the other two groups. Therefore, we propose that the postoperative histopathology for the diagnosis of CAM is not reliable in term pregnancy, and recommend that the diagnosis and delivery management should be based on clinical indicators. As stated by the NICHD expert panel, maternal fever alone should not automatically lead to the diagnosis of infection (or CAM) and antimicrobial treatment (Higgins et al., 2016). Meanwhile, researchers have searched for either antenatal or postnatal potential biomarkers to guide neonatal management. A recent meta-analysis suggested that there is insufficient evidence to support the use of CRP, procalcitonin, or IL6 in maternal blood for HCA diagnosis in PPROM (Etyang et al., 2020). As for other diagnostic indicators, Myntti et al. (2016) have demonstrated that increased lactate dehydrogenase and decreased blood glucose in amniotic fluid are connected with HCA by amniocentesis in 70 women with suspected intraamniotic infection. Another study has speculated that metalloproteinase (MMP-8) in amniotic fluid is a sensitive index for the diagnosis of microbial invasion of the amniotic cavity (Chaemsaithong et al., 2018). However, amniocentesis is an invasive procedure, which would also increase the risk of infection. Furthermore, given the correlation between genital tract microbiota and hormones, hormonal evaluation may also be a predictor of CAM. Except the positive correlation between E2 serum concentration and *Lactobacillus* abundance mentioned above, there are many other hormone and flora correlations reported in the literature. For example, *Prevotella* show a strong positive correlation with testosterone and negative associations with estradiol concentrations (Kumar, 2013); *Gemella* was detected as positively related with both FSH and LH (Sabo et al., 2020); a negative correlation was found between *Aerococcus*, *Atopobium* and LH hormone (Ling et al., 2010). Hence, we propose that parturients should be comprehensively evaluated for clinical symptoms, laboratory examination (WBC, CRP, or sex hormones), and fetal heart monitoring in the clinic.

It has long been thought that the diagnosis and progression of CAM were inextricably linked to infection, especially ascending infections. Previous studies have found that the placental microbiota is influenced by inflammation, with some oral and urogenital commensal bacteria playing a key role in CAM etiology (Prince et al., 2016). In a follow-up study, Hockney et al. (2020) confirmed that HCA is associated with infection and increased the bacterial load in a dose–response relationship. Common microorganisms in female genital tract infections include Group B *Streptococcus*, *E. coli*, *Bacillus*, *E. faecalis*, *U. urealyticum*, *M. hominis*, and bacterial vaginosis (Tibaldi et al., 2016). Moreover, several reports have shown that *Ureaplasma* spp. is the most prevalent pathogen in early-preterm birth or late-preterm (Sweeney et al., 2016; Matasariu et al., 2022). A prospective cohort study also detected *Mycoplasma and/or Ureaplasma* in 81% (29/36) of the vaginal secretions of preterm pregnant women (Paramel Jayaprakash et al., 2016). As can be seen from the samples, whether culture or 16S rRNA sequencing detected only a low percentage of positive rates. The most interesting finding was that the frequency of HCA is higher than that of CCA with positive bacterial cultures (37.8% in HCA group; 21.4% in CCA group). Therefore, we assume that histological and microbiological evidence of inflammation or infection may not always accompany one another. Similar to the above study, in the samples for which bacterial DNA could be detected, *Ureaplasma* spp. was higher in both the HCA and CCA groups than the normal group, especially in the CCA group. Furthermore, Romero et al. (2019) have conducted a cross-sectional study of women with intra-amniotic infection with intact membranes, and the results implied that the bacteria in the infected amniotic fluid were mainly *Sneathia*, *Ureaplasma*, *Prevotella*, *Lactobacillus*, *Escherichia*, *Gardnerella*, *Peptostreptococcus*, *Peptoniphilus* and *Streptococcus*, many of which were not cultured from the amniotic fluid samples. These results were highly similar to our current results for the intrauterine microbiota. The opportunistic pathogen *Ureaplasma* has been found in the urogenital tract and amniotic fluid of asymptomatic healthy and symptomatic women (Rumyantseva et al., 2019). Due to its high requirements for culture *in vitro* and slow growth, traditional culture detection is very complicated (van der Schalk et al., 2020). Thus, we hypothesized that this opportunistic pathogen not only causes premature labor but also plays a key role in term CAM. Hence, it is necessary to consider *Ureaplasma* infections when cephalosporin antibiotics are ineffective in CAM parturients in clinical practice, and a nucleic acid test should be used to improve the detection rate.

Moreover, Combs et al. (2014) have found that intra-amniotic inflammation is connected with adverse pregnancy outcomes regardless of the detection of microorganisms. In other words, despite the etiology, inflammation is a major driver of CAM. One retrospective study has shown that the term CCA is a syndrome that might be caused by intra-amniotic infection (63%) and sterile intra-amniotic inflammation (5%). Additionally, a considerable number of patients have no evidence of intraamniotic infection or inflammation (Romero et al., 2021). Besides, Roberts et al. (2012) have suggested that CAM might be a non-infectious inflammatory process. Thus, the activation of the maternal immune state at term might lead to similar changes in the placenta. Infection is inflammation caused by the invasion of bacteria, viruses, fungi or other pathogens, while inflammation is a basic pathological process in which the tissue with vascular system reacts to various injury factors. Increasing evidence has shown that inflammation without microorganisms might be elicited by activation of endogenous danger signals derived from cellular stress or necrosis, known as damage-associated molecular patterns (DAMPs) or alarmins (Romero et al., 2011). Furthermore, a recent study compared sterile intra-amniotic inflammation and those with intra-amniotic infection *via* RNA sequencing, confirm that the immune response in sterile inflammation is notably different and milder than microbial-induced inflammation (Motomura et al., 2021). Nevertheless, to date, there is no approved treatment for sterile intra-amniotic inflammation. Meanwhile, Galaz et al. (2021) used a mouse model of HMGB1-induced sterile inflammation and demonstrated that betamethasone treatment prevents preterm birth but does not reduce neonatal mortality. In addition, a recent study had proved that intravenous clarithromycin could reduce the intensity of sterile inflammation in patients with PPROM by monitoring IL-6 concentrations (Kacerovsky et al., 2020). Future studies should consider the mechanisms of intrauterine inflammation and focus on the treatment of sterile inflammation.

In current study, we not only analyzed the intrauterine microbiota in normal and CAM patients but also comprehensively considered its trend in the upper and lower genital tracts from a macroscopic perspective. However, our research also has some limitations. First, negative control samples (operating room environment, surgical instruments, and DNA extraction kits) were not subjected to 16S sequencing because no DNA of acceptable quality was extracted. Second, our sample size was small and experiments with larger samples are required to further prove our current results.

## Conclusion

In summary, some normal-term pregnant women can harbor non-pathogenic microbiota in the uterine cavity. Additionally, sterile inflammation is more frequent than microbial-associated inflammation in full-term HCA and CCA patients. Besides, we recommend a combined evaluation of clinical symptoms, laboratory examination, and FHR monitoring to diagnose CAM at term. Finally, it is necessary to determine a sensitive biomarker in the diagnosis of CAM in future studies.

## Data availability statement

The data presented in the study are deposited in the NCBI repository, accession number PRJNA904128.

## Ethics statement

The studies involving human participants were reviewed and approved by Ethics Committee of Beijing Tsinghua Changgung Hospital (16121-0110). The patients/participants provided their written informed consent to participate in this study. Written informed consent was obtained from the individual(s) for the publication of any potentially identifiable images or data included in this article.

## Author contributions

ML and ZH: conceptualization and writing – original draft. ZT, YM, and JW: data curation and formal analysis. QZ, MS, and YL: funding acquisition, investigation, and methodology. YiW, YuW, XW, YC, and RC: project administration, resources, and software. QL and LZ: supervision, validation, visualization, and writing – reviewing and editing. All authors contributed to the article and approved the submitted version.

## Funding

Tsinghua University Chunfeng Fund domestic research Project (405-10001000221) 2021Z99CFY032; Key Laboratory of Precision Medicine, Tsinghua University (20219990012); National Natural Science Foundation of China, Key instrument Research and development Project (61927819); Beijing Natural Fund Committee, general project (7202239).

## Conflict of interest

The authors declare that the research was conducted in the absence of any commercial or financial relationships that could be construed as a potential conflict of interest.

## Publisher’s note

All claims expressed in this article are solely those of the authors and do not necessarily represent those of their affiliated organizations, or those of the publisher, the editors and the reviewers. Any product that may be evaluated in this article, or claim that may be made by its manufacturer, is not guaranteed or endorsed by the publisher.
